# Association of Cystatin C with Metabolic Syndrome and Its Prognostic Performance in Non-ST-Segment Elevation Acute Coronary Syndrome with Preserved Renal Function

**DOI:** 10.1155/2019/8541402

**Published:** 2019-06-16

**Authors:** Qi Mao, Ning Zhao, Yuqing Wang, Youmei Li, Chaojun Xiang, Lufeng Li, Wei Zheng, Shangcheng Xu, Xiao-Hui Zhao

**Affiliations:** ^1^Department of Cardiovascular Medicine, Institute of Cardiovascular Research, Xinqiao Hospital, Army Medical University, Chongqing 400037, China; ^2^Department of Occupational Health, Army Medical University, Chongqing 400038, China

## Abstract

**Objective:**

The underlying mechanisms by which cystatin C affects cardiovascular disease (CVD) are not very clear. Metabolic syndrome (MetS) is a cluster of risk factors that increase the risk of CVD. Here, we aimed to investigate the association of cystatin C with metabolic syndrome and cardiovascular outcomes in non-ST-segment elevation acute coronary syndrome (NSTE-ACS) with preserved renal function.

**Methods:**

In total, 422 NSTE-ACS patients with preserved renal function were enrolled to examine the association of cystatin C with MetS. MetS was defined based on the NCEP-ATP-III guidelines. Major adverse cardiovascular events (MACEs) were also evaluated, which included cardiac death, nonfatal myocardial infarction (MI), target vessel revascularization (TVR), heart failure, and nonfatal stroke. All patients underwent a 12-month follow-up for MACEs after admission.

**Results:**

Cystatin C was significantly correlated with metabolic risk factors and inflammation markers. The prevalence of MetS and MACEs correlated with cystatin C levels. Cystatin C showed a strong diagnostic performance for cardiovascular risk factors and outcomes in ROC analysis. After adjustment for multiple risk factors, cystatin C level was independently associated with MetS (OR 2.299, 95% CI 1.251–4.225, and P = 0.007). During a 12-month follow-up, the patients with high cystatin C level and MetS had higher incidence of MACEs (Log-rank = 24.586, P < 0.001) and cardiac death (Log-rank = 9.890, P = 0.020) compared to the others. Multivariate Cox analysis indicated that cystatin C level was an independent predictor of MACEs (HR 2.609, 95% CI 1.295–5.257, and P = 0.007).

**Conclusion:**

Cystatin C may be an independent predictor of metabolic syndrome and therefore valuable for management of NSTE-ACS patients. Further multicenter, large-scale studies are required to assess the implication of these results.

## 1. Introduction

Cystatin C is an endogenous inhibitor of cathepsin cysteine proteases and is generally considered to be constantly secreted and be freely filtered by the glomerulus but be neither secreted by the renal tubule nor reabsorbed into circulation [1, 2]. Therefore, cystatin C is useful for estimation of glomerular filtration rate (GFR) and known as a marker of renal function [3, 4].

Recent studies have described cystatin C as a prominent predictor of cardiovascular diseases (CVD) that is significantly associated with high risk of cardiovascular outcomes in acute coronary syndrome (ACS) [5, 6]. Until now, the reasons by which cystatin C is associated with cardiovascular outcomes were mostly attributed to its higher sensitivity for identifying early renal impairment [7–9]. However, increasing evidence indicated that cystatin C not only was a marker of GFR but also was correlated with inflammation and oxidative stress in CVD [10–12]. The Prospective Epidemiological Study of Myocardial Infarction (PRIME) showed that cystatin C was associated with coronary events independent of estimated GFR [13]. Tangri et al. [14] also reported that cystatin C remained associated with cardiovascular events even after adjustment for directly measured GFR. These results indicated that cystatin C was a predictor of cardiovascular events independent of renal function and implied that non-GFR determinants of cystatin C might be related to cardiovascular outcomes.

Of note, several studies have reported that hypertension, dyslipidemia, and diabetes were associated with cystatin C level, which were the components of metabolic syndrome (MetS) and cardiometabolic risk factors [15–17]. Interestingly, recent cross-sectional studies also showed that cystatin C level increased in patients with MetS and may be used as a marker of MetS in general population [18–20]. MetS, characterized by glucose and lipid disorder, is an important risk factor for ACS and is an increasing epidemic worldwide [21–23]. To the best of our knowledge, the relationship between cystatin C and metabolic risk factors remains unclear in ACS, and no studies have explored the association of cystatin C with MetS in NST-ACS. We hypothesized that cystatin C might be associated with MetS independent of renal function and therefore aimed to investigate the role of non-GFR determinants of cystatin C on cardiovascular risk factors and major adverse cardiovascular events (MACEs) in NSTE-ACS.

## 2. Materials and Methods

### 2.1. Study Population

The study protocol complied with the Declaration of Helsinki and was approved by Xinqiao Hospital Ethics Committee, Army Military Medical University (Chongqing, China). All patients provided informed consent.

This was a prospective observational study consisting of 797 consecutive patients with NSTE-ACS patients with preserved renal function who were admitted between January 2017 and September 2017. The inclusion criteria were as follows: (1) with complete clinical information; (2) all patients underwent coronary angiography; and (3) preserved renal function defined as estimated glomerular filtration rate (eGFR) ≥ 60 mL/min *∗* 1.73 m^2^ at admission. The exclusion criteria were nonobstructive coronary disease, primary cardiomyopathy and valvular heart disease, primary kidney disease, severe hepatic dysfunction, significant infection, thyroid and adrenal cortex dysfunction, autoimmune diseases, use of steroids and immune inhibitors, hematologic disorders, surgery or trauma 3 month prior to participation, and malignant diseases. Finally, a cohort of 422 patients was enrolled in this study.

### 2.2. Data Collection and Follow-up

Clinical data were collected from medical records by trained physicians. These included demographic data, medical history, laboratory parameters, and basic medication information. The venous blood samples were collected after overnight fasting before coronary angiography, and routine biochemical indicators were measured by automatic biochemical analyzer (DXC800, Beckman Coulter, USA). Concentration of serum cystatin C was determined by particle-enhanced turbidimetric immunoassay (PETIA) method. The angiographic data were obtained from the cardiac catheterization laboratory records. The synergy between percutaneous coronary intervention with TAXUS and cardiac surgery (SYNTAX) scores for quantifying coronary lesions was assessed by experienced interventional cardiologists using the score calculator (version 2.28) in SYNTAX score website. Primary outcomes were major adverse cardiovascular events (MACEs) defined as the combination of cardiac death, nonfatal myocardial infarction, target vessel revascularization (TVR), heart failure, and nonfatal stroke [24]. All patients had 12-month follow-up after admission, and follow-up data were obtained from hospital records (39 cases, 9.2%) or by interviewing (in person or by telephone) patients (369 cases, 87.5%) and their families (14 cases, 3.3%).

### 2.3. Definition

The definition of NSTE-ACS complied with the current guidelines of the European Society of Cardiology (ESC) [25]. Global Registry of Acute Coronary Events (GRACE) risk score was applied to stratification and prediction of risk in patients with ACS and was calculated based on the clinical history, electrocardiogram, and laboratory parameters at admission [26]. Multivessel disease was a type of complex coronary artery disease associated with poor prognosis, defined as at least double-vessel disease or left main disease with > 50% luminal narrowing [27]. SYNTAX score was a clinical tool for quantifying coronary lesions and was dependent of all coronary lesions with > 50% diameter stenosis in a vessel > 1.5 mm [28]. The basic drug treatment for the NSTE-ACS patients was in compliance with the current ESC guidelines [25]. Percutaneous coronary intervention (PCI) was a conventional treatment strategy for revascularization and reperfusion in patients with ACS and was determined by experienced cardiologists based on individual risk and decisions from patients. Metabolic syndrome (MetS) was a cluster of metabolic disorders associated with CVD and its definition complied with National Cholesterol Education Program's Adult Treatment Panel III (NCEP ATP III) criteria [29]. Estimated glomerular filtration rate (eGFR) reflected renal impairment and was calculated based on the Modification of Diet in Renal Disease (MDRD) Study equation: eGFR (mL/min *∗* 1.73 m^2^) = 186 × Scr^−1.154^× age^−0.203^× 1.233 × 0.742 (if female) [30]. C-reactive protein (CRP) and B-type natriuretic peptide (BNP) were markers of inflammation and heart failure, respectively. Both of them were, respectively, converted into binary categorical variables by 5 *μ*g/mL as the cutoff value of elevated CRP level and 100 pg/mL as the cutoff value of elevated BNP level [31, 32]. Killip class was used for clinical grading of heart failure caused by acute myocardial infarction. Killip class > 1 was considered as elevated Killip class. SYNTAX ≥ 23 and GRACE ≥ 89 were, respectively, defined as a high SYNTAX score and a high GRACE score [26, 28].

### 2.4. Statistical Analysis

Continuous variables were expressed as mean ± SD or median (IQR) according to the presence or absence of normal distribution, and categorical data were expressed as frequencies and percentages. To compare the baseline characteristics, the study population was divided into two groups by the median cystatin C level (0.90 mg/L) of the cohort: low cystatin C group (≤ 0.90 mg/L) and high cystatin C group (> 0.90 mg/L). The* t* test was used if continuous variables were normally distributed, while the Mann–Whitney U test was applied if continuous variables were not normally distributed. Differences in categorical variables were evaluated by the Chi-squared test or Fisher exact test. Correlation was assessed using the Spearman rank correlation test. Diagnostic performances of cystatin C on cardiovascular risk factors and outcomes were assessed by the receiver operating characteristic (ROC) curve analysis. Logistic regression was applied to explore the association between cystatin C and MetS, and the variables with unadjusted P value of < 0.1 were selected as potential risk factors and included in the multivariate model. Event-free survival time was defined as from the date of admission to the date of cardiovascular events as verified during the follow-up. Survival curves or cumulative risk curves for cardiovascular outcomes were constructed using the Kaplan-Meier method, with differences assessed using the log-rank test. Cox regression analyses were performed to evaluate the association of cystatin C with MACEs. Multivariate Cox models were adjusted for established cardiovascular risk factors (age, gender, smoking, hypertension, diabetes, LDL-C, and HDL-C), clinical variables affecting cardiovascular outcomes (Killip class, GRACE scores, and SYNTAX scores), medication (ACEI/ARB, *β*-blocker, and PCI/CABG), and variables that might affect cystatin C levels (BMI, eGFR, and CRP). Adjusted model 1 included age, gender, BMI, smoking, hypertension, diabetes, LDL-C, and HDL-C; and adjusted model 2 same as model 1 plus eGFR, CRP, Killip class > 1, GRACE scores, SYNTAX scores, ACEI/ARB, *β*-blocker, and PCI/CABG. P < 0.05 was considered statistically significant. All statistical analyses were performed using SPSS software version 22.0 (SPSS, Inc., Chicago, Illinois).

## 3. Results

### 3.1. Baseline Characteristics of the Study Population

The characteristics of the study population are described in Table 1. Patients in the high cystatin C group were older than those in the low cystatin C group (P < 0.001). The patients in the high cystatin C group had a higher incidence of diabetes (P = 0.036) and MetS (P = 0.001), had more severe coronary artery lesion (P < 0.001), and had a higher GRACE score (P < 0.001). Significant differences in clinical laboratory parameters were also observed in the two groups.

### 3.2. Correlation of Cystatin C, Creatinine, and eGFR-MDRD with Other Clinical Variables

Spearman rank correlation analysis was used to examine the correlation of cystatin C, creatinine and eGFR-MDRD with other clinical variables. Compared to creatinine and eGFR-MDRD, cystatin C showed greater correlation with metabolic parameters, inflammation markers, and other cardiovascular risk factors (Table 2).

### 3.3. Comparison of Cardiovascular Risk Factors and Events according to Restratification Based on Cystatin C Level and Metabolic Syndrome

**Figure 1:**
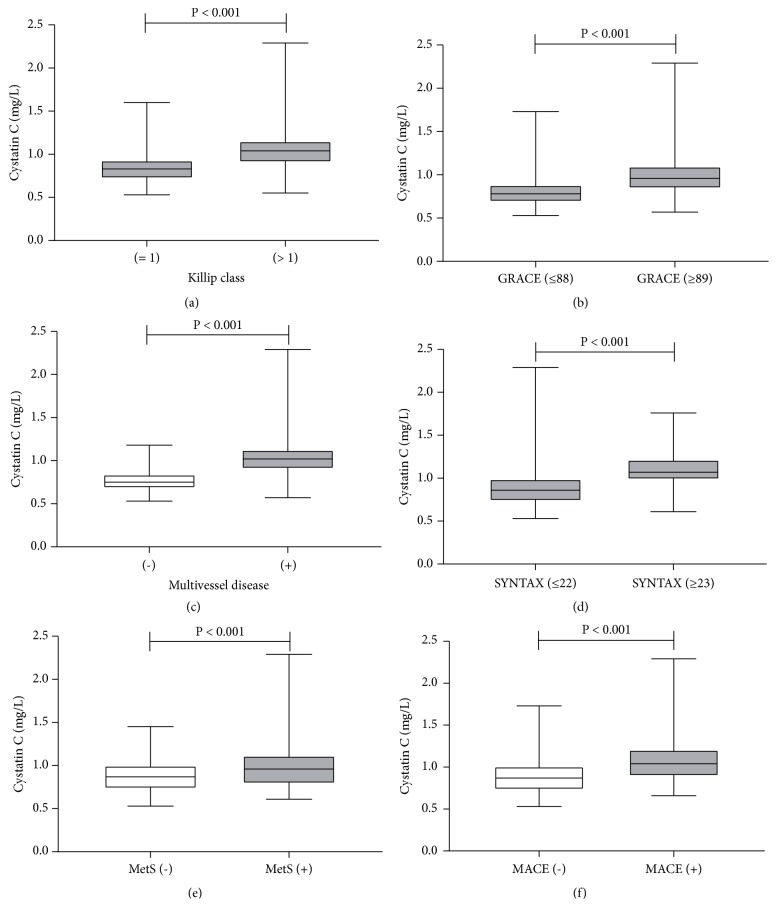
*Comparisons of cystatin C levels in NSTE-ACS with or without Killip class > 1 (a), high GRACE score (b), multivessel disease (c), high SYNTAX score (d), metabolic syndrome (e), and MACE (f) by using the Mann–Whitney U test.* MetS: metabolic syndrome; MACE: major adverse cardiovascular events.

Furthermore, we stratified the subjects with or without MetS into the following four groups based on cystatin C levels: low cystatin C/MetS (-) (n = 160), low cystatin C/MetS (+) (n = 51), high cystatin C/MetS (-) (n = 128), and high cystatin C/MetS (+) (n = 83). Elevated CRP level, elevated BNP level, elevated Killip class, high GRACE score, multivessel disease, high SYNTAX score, and MACEs were significantly different among the four groups (P < 0.001), and the high cystatin C/MetS (+) group had more cardiovascular risk factors and events than the other three groups (Table 3). In addition, comparisons of baseline cystatin C levels in the NSTE-ACS patients with or without Killip class > 1, multivessel disease, high SYNTAX score, high GRACE score, MetS, and MACEs are shown in Figure 1.

### 3.4. Diagnostic Performance of Cystatin C for Cardiovascular Risk Factors and Outcomes

In ROC curve analysis, the predictive cutoff values of cystatin C were constructed according to the ROC curves for identifying the patients with more cardiovascular risk factors, and for predicting the occurrence of MACEs. According to the area under the curve (AUC), cystatin C showed a powerful diagnostic performance for cardiovascular risk factors and outcomes. Using the cutoff points, the predictive values of cystatin C for MetS and MACEs were 1.01 mg/L and 0.87 mg/L, respectively (Table 4).

### 3.5. Association of Cystatin C with Metabolic Syndrome

Potential risk factors for metabolic syndrome were chosen in univariate logistic regression analysis (P < 0.1) and were used as the variables of multivariate model. The results indicated that cystatin C level was independently associated with metabolic syndrome (OR 2.299, 95% CI 1.251–4.225, and P = 0.007) (Table 5).

### 3.6. Association of Cystatin C with Cardiovascular Outcomes

**Figure 2:**
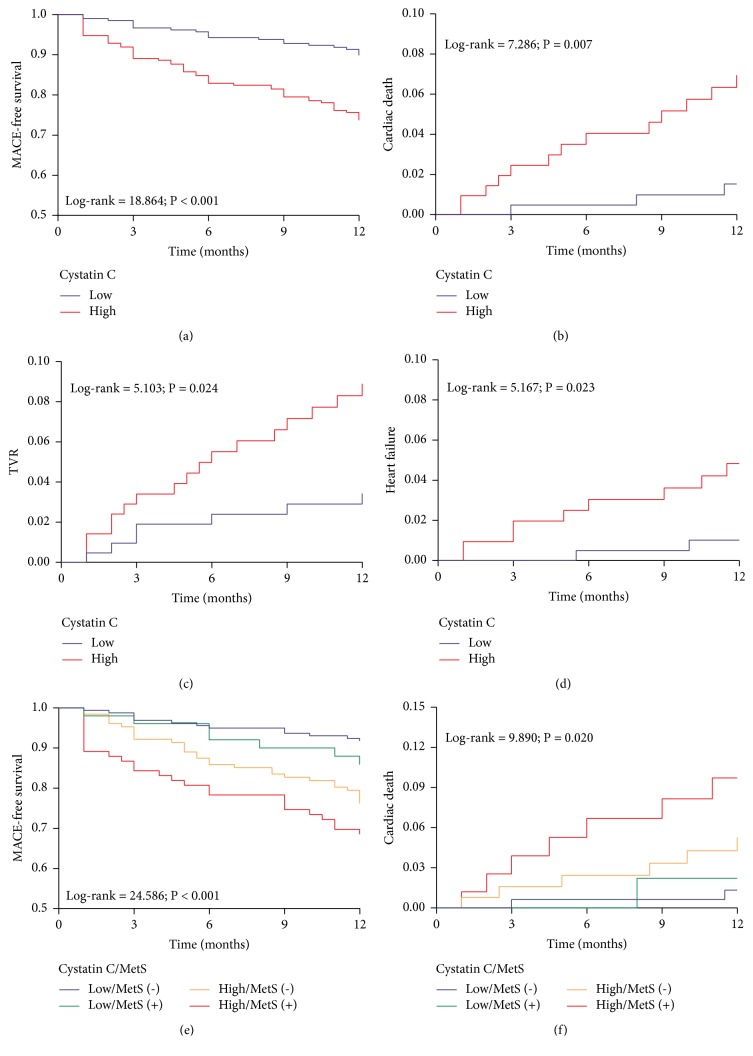
*Kaplan-Meier curves for survival analysis of MACE-free (a, e); cumulative hazard curves for cardiac death (b, f), TVR (c), and heart failure (d).* Significant differences between groups were assessed by the log-rank test. MACE: major adverse cardiovascular events; TVR: target vessel revascularization.

Cox proportional hazard model was used to examine the association between cystatin C and cardiovascular outcomes during the 12-month follow-up. Univariate analysis showed that cystatin C was significantly associated with MACEs and its components. Confounders were included in the multivariate model for adjustment, and cystatin C remained to be an independent predictor of MACEs in adjusted models 1 (HR 2.677, 95% CI 1.566–4.576, and P < 0.001) and 2 (HR 2.609, 95% CI 1.295–5.257, and P = 0.007) (Table 6). In the Kaplan-Meier analysis, the incidence of MACEs (Log-rank = 18.864, P < 0.001), cardiac death (Log-rank = 7.286, P = 0.007), TVR (Log-rank = 5.103, P = 0.024), and heart failure (Log-rank = 5.167, P = 0.023) was higher in the high cystatin C group than that in the low cystatin C group. Moreover, the high cystatin C/MetS (+) group had the higher incidence of MACEs (Log-rank = 24.586, P < 0.001) and cardiac death (Log-rank = 9.890, P = 0.020) than the other three groups (Figure 2).

## 4. Discussion

Our study focused on the link between non-GFR determinants of cystatin C and cardiometabolic risk factors and highlighted the predictive role of non-GFR determinants in NSTE-ACS. To the best of our knowledge, we for the first time examined the relationship between cystatin C and MetS in NSTE-ACS with preserved renal function. In our study, the main findings were as follows: (1) cystatin C was a powerful diagnostic indicator for cardiovascular risk factors; (2) cystatin C level was independently associated with MetS; and (3) cystatin C level was an independent predictor of MACEs during 12-month follow-up.

Renal impairment reflected by decreased GFR is known as an important cardiovascular risk factor [33]. Considering that direct measurement of GFR is cumbersome and not conductive to clinical application, renal function is mainly evaluated by estimated GFR (eGFR) based on the level of creatinine or cystatin C [3]. As circulating creatinine consists of GFR determinants and non-GFR determinants, circulating concentration of cystatin C has also been reported not to be entirely dependent on filtration rate, and its expression and secretion are regulated by several potential mechanisms [10, 34]. Recent studies further indicated that non-GFR determinants of cystatin C participated in many physiological processes and played an important role in vascular biology [35]. Elevated circulating cystatin C level not only represents reduced GFR but also may reflect the compensation for increased elastolytic activity and the response to increased inflammatory factors and oxidative stress [11, 16]. Hence, the prominent predictive effect of cystatin C on CVD should not simply interpreted by the filtration rates. However, until now the relationship between non-GFR determinants of cystatin C and cardiovascular risk factors had been controversial, and the potential mechanisms involved in cardiovascular outcomes were unclear [36].

Previous evidence revealed the significant association between cystatin C and metabolic risk factors, such as dyslipidemia, diabetes, and hypertension [19, 20, 37]. Our findings provided further insight into the potential link between cystatin C and MetS in NSTE-ACS. First, our results showed the correlation of cystatin C with metabolic parameters and inflammation markers rather than creatinine. Second, we observed that patients with higher cystatin C levels had worsened conditions of MetS compared to those with low cystatin C levels. Consistent with our results, Surenda et al. [19] noted that cystatin C level increased with increasing number of metabolic abnormalities in the Asian population with normal glucose tolerance; and Servais et al. [38] reported that cystatin C was significantly higher in patients with MetS independent of creatinine level. However, these studies were mainly based on general population, as until now no studies have been focused solely on patients with ACS. Hence, our results were complementary to previous observations. Third, the ROC curve analysis indicated that cystatin C might serve as a diagnostic indicator for MetS. The results reconfirmed the correlation with MetS and suggested that non-GFR determinants of cystatin C might participate in the process of metabolic disorder. In this context, elevated cystatin C level may contribute to identify the patients with MetS for risk stratification.

In previous studies, Magnusson et al. [18] reported that cystatin C might contribute to development of MetS by affecting metabolic parameters in general population, and Vigil et al. [20] showed that circulating cystatin C included several non-GFR determinants by multiple linear regression analysis in a hypertension population with a larger proportion of MetS. In the present study, we found that cystatin C remained to be an independent predictor of MetS even after adjustment for eGFR and other risk factors in multivariate analysis. The independent effect of cystatin C on MetS might be attributed to metabolic factors related with cystatin C. A few studies showed that high cystatin C level was associated with central adiposity characterized by visceral fat accumulation [39–41]. Further evidence suggested that cathepsin/cystatin system regulated the differentiation of preadipocyte and adipocyte to promote fat accumulation [42, 43]. Recent studies also indicated that the relationship between cystatin C and diabetes was dependent of central adiposity, and cystatin C was significantly associated with IR independent of filtration rate [44, 45]. Visceral fat accumulation is known to be strongly associated with insulin resistance (IR) that contributes to the pathogenesis of MetS [21, 46]. Thus, high cystatin C level may reflect visceral fat accumulation and increased insulin resistance. Furthermore, human tissue biopsy data revealed that both cathepsin and cystatin C were highly expressed in adipose tissue inflammation, which is closely related to IR and MetS [39]. Due to the inhibition of cathepsin by cystatin C, these findings suggested that cystatin C might contribute to controlling adipose tissue homeostasis and to inhibiting cathepsin-induced inflammation by countering the activation of cathepsin [41]. Therefore, elevated non-GFR determinants of cystatin C may represent the response to adipose tissue remodeling and the progression of insulin resistance. The relationship of cystatin C with MetS may be further associated with IR dependent on non-GFR determinants of cystatin C. Moreover, IR reflected by cystatin C could also independently elucidate the association of cystatin C with other cardiovascular risk factors, such as SYNTAX score and GRACE score, in NSTE-ACS with preserved renal function.

In the current study, we found that cystatin C was a strong independent predictor of MACEs in NSTE-ACS, even after adjustment for CRP, MetS, eGFR, and SYNTAX scores. The results indicated that non-GFR determinants of cystatin C were associated with cardiovascular outcomes, which was supported by previous studies that directly measured GFR thus avoiding errors of eGFR [47, 48]. Similarly, the Ludwigshafen Risk and Cardiovascular Health (LURIC) study also demonstrated that cystatin C was predictive for all-cause and cardiovascular mortality independent of renal function [49]. Hence, the predictive role of cystatin C could not be simply ascribed to renal impairment but further extended to functions in vascular remodeling and plaque instability. Therefore, the underlying mechanisms by which non-GFR determinants of cystatin C affect cardiovascular outcomes may be as follows: first, cystatin C may induce inflammation and promote the progression of atherosclerosis [50]. Second, cystatin C may directly mediate the degradation of vascular matrix, remodel coronary artery wall, and reconstruct plaque [51]. Third, IR reflected by cystatin C may contribute to plaque instability [52]. The rupture of unstable plaque was the leading cause of acute myocardial infarction, increased plaque burden determined ischemic-driven revascularization, and finally continuous myocardial ischemia and ventricular remodeling greatly increased risk of cardiovascular death [23, 53]. In addition, IR reflected by cystatin C may also promote sympathetic activity, activate the renin-angiotensin-aldosterone system, and ultimately increase the incidence of MACEs [54, 55].

Our study had several limitations. Firstly, this was a single center and observational study with potential selection bias. Second, our sample size was relatively small, and follow-up duration was short. Third, laboratory parameters were only measured once at admission with potential bias due to measurement error. Fourth, our study failed to rely on direct GFR measurement to assess the association of GFR with cardiovascular risk factors and outcomes that could not completely exclude the compounded effect of GFR on the predictive role of cystatin C [11]. Fifth, IR and MetS are considered to be associated with glomerular hyperfiltration in early renal impairment and may increase cystatin C level due to IR underestimated hyperfiltration [56]. Finally, since our study population is of Chinese origin, further population-based studies are required before extrapolating the findings of this study to make a general conclusion.

## 5. Conclusion

Our study indicated that non-GFR determinants of cystatin C might be an independent predictor of MetS and cardiovascular outcomes in NSTE-ACS. Cystatin C links renal impairment, insulin resistance, vascular remodeling, and cardiovascular risk as a cardiorenal metabolic marker. The detection of cystatin C may be helpful for management of NSTE-ACS to decrease cardiovascular events. Given the limitations of current study, further multicenter, large-scale, long follow-up period studies are required to assess the implication of these results.

## Figures and Tables

**Table 1:** Baseline characteristics of the study population.

Variables	Total	Cystatin C		*P* value
	N = 422	Low (≤ 0.90 mg/L, n = 211)	High (> 0.90 mg/L, n = 211)	
Age(years)	60.51 ± 10.07	57.47 ± 10.08	63.55 ± 9.12	<0.001
Male, n (%)	283 (67.1)	142 (67.3)	141 (66.8)	0.918
BMI (kg/m^2^)	24.25 ± 3.26	24.40 ± 3.33	24.10 ± 3.20	0.336
Smoking, n (%)	187 (44.3)	91 (43.1)	96 (45.5)	0.624
Hypertension, n (%)	132 (31.3)	70 (33.2)	62 (29.4)	0.401
Diabetes, n (%)	132 (31.3)	56 (26.5)	76 (36.0)	0.036
Stroke history, n (%)	45 (10.7)	24 (11.4)	21 (10.0)	0.636
Metabolic syndrome, n (%)	134 (31.8)	51 (24.2)	83 (39.3)	0.001
Killip class > 1, n (%)	162 (38.4)	39 (18.5)	123 (58.3)	<0.001
Multivessel disease, n (%)	245 (58.1)	58 (27.5)	187 (88.6)	<0.001
SYNTAX score	11.0 (7.0–18.0)	7.0 (4.0–11.0)	16.5 (12.0–23.0)	<0.001
GRACE score for 6 months	100.0 (81.0–122.0)	85.0 (70.0–101.0)	117.0 (98.0–132.0)	<0.001
*Medication*				
ACEI/ARB, n (%)	355 (84.1)	175 (82.9)	180 (85.3)	0.505
Beta-blocker, n (%)	327 (77.5)	161 (76.3)	166 (78.7)	0.560
PCI/CABG, n (%)	346 (82.0)	174 (82.5)	172 (81.5)	0.800
*Laboratory parameters*				
TG (mmol/L)	1.44 (1.01–2.01)	1.31 (0.95–1.94)	1.48 (1.08–2.11)	0.020
LDL-C (mmol/L)	2.72 ± 0.81	2.70 ± 0.79	2.73 ± 0.82	0.723
HDL-C (mmol/L)	1.01 (0.85–1.18)	1.02 (0.86–1.22)	1.00 (0.85–1.17)	0.295
TG/HDL-C ratio	1.416 (0.949–2.201)	1.320 (0.882–1.972)	1.511 (1.007–2.279)	0.009
FBG (mmol/L)	5.15 (4.60–6.16)	4.98 (4.56–5.90)	5.30 (4.62–6.35)	0.067
HbA1c (%)	6.0 (5.7–6.6)	5.9 (5.6–6.4)	6.1 (5.8–6.7)	0.011
Cystatin C (mg/L)	0.90 (0.76–1.04)	0.76 (0.69–0.83)	1.04 (0.96–1.15)	<0.001
Uric acid (*μ*mol/L)	339.7 (283.4–405.8)	327.3 (266.0–388.6)	357.6 (306.7–426.5)	<0.001
Creatinine (*μ*mol/L)	72.6 ± 14.8	67.6 ± 13.3	77.6 ± 14.6	<0.001
eGFR-MDRD (mL/min *∗* 1.73 m^2^)	96.1 (80.6–108.1)	101.4 (91.4–117.4)	86.2 (74.8–100.9)	<0.001
BNP (pg/mL)	69.7 (21.0–183.0)	46.5 (12.9–116.0)	102.5 (39.5–314.3)	<0.001
WBC (10^9^/mL)	6.75 (5.64–8.43)	6.71 (5.61–8.30)	6.76 (5.75–8.81)	0.604
Neutrophil (10^9^/mL)	4.33 (3.33–5.88)	4.23 (3.28–5.54)	4.47 (3.39–6.34)	0.226
Monocyte (10^9^/mL)	0.52 (0.39–0.66)	0.48 (0.38–0.64)	0.54 (0.43–0.67)	0.064
Lymphocyte (10^9^/mL)	1.57 (1.23–1.96)	1.63 (1.30–2.08)	1.50 (1.16–1.83)	0.002
NLR	2.77 (1.90–4.12)	2.61 (1.78–3.63)	3.00 (2.04–4.51)	0.004
MLR	0.33 (0.24–0.45)	0.29 (0.23–0.40)	0.35 (0.27–0.49)	<0.001
CRP (*μ*g/mL)	6.25 (5.00–11.03)	5.00 (5.00–6.00)	10.30 (7.03–17.50)	<0.001
D-dimer (mg/L)	0.26 (0.15–0.51)	0.22 (0.14–0.46)	0.28 (0.16–0.63)	0.020
Fibrinogen (g/L)	2.91 (2.45–3.60)	2.76 (2.32–3.51)	3.08 (2.57–3.72)	0.004

Data are expressed as mean±SD, median (IQR) or n (%).

*Abbreviations.* BMI: body mass index; ACEI: angiotensin converting enzyme inhibitors; ARB: angiotensin receptor blocker; PCI: percutaneous coronary intervention; CABG: coronary artery bypass grafting; TG: triglyceride; LDL-C: low-density lipoprotein cholesterol; HDL-C: high-density lipoprotein cholesterol; TG/HDL-C ratio: triglyceride to high-density lipoprotein cholesterol ratio; FBG: fasting blood glucose; eGFR-MDRD: estimated glomerular filtration rate based on MDRD equation; BNP: B-type natriuretic peptide; WBC: white blood cell; NLR: neutrophil to lymphocyte ratio; MLR: monocyte to lymphocyte ratio; CRP: C-reaction protein.

**Table 2:** Correlation of cystatin C, creatinine, and eGFR-MDRD with Other Clinical Variables.

	Cystatin C		Creatinine		eGFR-MDRD	
Variables	*r*	*P* value	*r*	*P* value	*r*	*P* value
Age	0.383	<0.001	0.022	0.651	-0.352	<0.001
TG/HDL-C ratio	0.133	0.006	0.110	0.024	-0.035	0.473
TG	0.109	0.025	0.055	0.261	-0.061	0.209
FBG	0.107	0.028	0.001	0.990	-0.023	0.631
HbA1c	0.128	0.008	0.016	0.740	-0.087	0.074
Uric acid	0.237	<0.001	0.455	<0.001	-0.309	<0.001
Creatinine	0.394	<0.001	–	–	–	–
eGFR-MDRD	-0.469	<0.001	-0.752	<0.001	–	–
BNP	0.328	<0.001	0.125	0.010	-0.199	<0.001
GRACE score	0.593	<0.001	0.207	<0.001	-0.390	<0.001
SYNTAX score	0.597	<0.001	0.247	<0.001	-0.319	<0.001
Monocyte	0.101	0.038	0.140	0.004	-0.034	0.490
Lymphocyte	-0.165	0.001	-0.057	0.240	0.045	0.352
NLR	0.156	0.001	0.079	0.106	-0.040	0.416
MLR	0.217	<0.001	0.188	<0.001	-0.065	0.182
CRP	0.641	<0.001	0.279	<0.001	-0.334	<0.001
D-dimer	0.166	0.001	0.034	0.486	-0.178	<0.001
Fibrinogen	0.181	<0.001	0.069	0.157	-0.039	0.420

*Abbreviations.* TG: triglyceride; HDL-C: high-density lipoprotein cholesterol; TG/HDL ratio: triglyceride to high-density lipoprotein cholesterol ratio; FBG: fasting blood glucose; eGFR-MDRD: estimated glomerular filtration rate based on MDRD equation; BNP: B-type natriuretic peptide; NLR: neutrophil to lymphocyte ratio; MLR: monocyte to lymphocyte ratio; CRP: C-reactive protein.

**Table 3:** Cardiovascular risk factors and follow-up cardiac events.

	Low cystatin C (≤ 0.90 mg/L, n = 211)		High cystatin C (> 0.90 mg/L, n = 211)		
	MetS (-), n = 160	MetS (+), n = 51	MetS (-), n = 128	MetS (+), n = 83	*P* value
CRP > 5 *μ*g/mL, n (%)	55 (34.4)	28 (54.9)	103 (80.5)	76 (91.6)	<0.001
BNP > 100 pg/mL, n (%)	44 (27.5)	17 (33.3)	64 (50.0)	43 (51.8)	<0.001
Killip class > 1, n (%)	30 (18.8)	9 (17.6)	74 (57.8)	49 (59.0)	<0.001
Multivessel disease, n (%)	42 (26.3)	16 (31.4)	108 (84.4)	79 (95.2)	<0.001
GRACE score ≥ 89, n (%)	65 (40.6)	24 (47.1)	109 (85.2)	70 (84.3)	<0.001
SYNTAX score ≥ 23, n (%)	5 (3.1)	2 (3.9)	31 (24.2)	27 (32.5)	<0.001
MACEs, n (%)	13 (8.1)	7 (13.7)	30 (23.4)	26 (31.3)	<0.001
Cardiac death, n (%)	2 (1.3)	1 (2.0)	6 (4.7)	7 (8.4)	0.036
Non-fatal MI, n (%)	3 (1.9)	3 (5.9)	8 (6.3)	6 (7.2)	0.116
TVR, n (%)	4 (2.5)	3 (5.9)	9 (7.0)	8 (9.6)	0.086
Heart failure, n (%)	2 (1.3)	0 (0)	5 (3.9)	4 (4.8)	0.182
Non-fatal stroke, n (%)	2 (1.3)	0 (0)	2 (1.6)	1 (1.2)	1.000

Data are expressed as n (%). *P* value is from Chi-square test or Fisher exact test.

*Abbreviations.* CRP: C-reaction protein; BNP: B-type natriuretic peptide; MACEs: major adverse cardiovascular events; MI: myocardial Infarction; TVR: target vessel revascularization; MetS: metabolic syndrome.

**Table 4:** Summary of ROC curves.

	AUC	95% CI	*P* value	Cys C cutoff (mg/L)	Sensitivity	Specificity	Youden index
CRP > 5 *μ*g/mL	0.793	(0.749–0.837)	<0.001	0.85	0.782	0.719	0.501
BNP > 100 pg/mL	0.658	(0.605–0.712)	<0.001	1.02	0.464	0.811	0.275
Killip class > 1	0.774	(0.727–0.822)	<0.001	0.93	0.722	0.742	0.465
Multivessel disease	0.887	(0.858–0.919)	<0.001	0.91	0.751	0.887	0.638
SYNTAX score ≥ 23	0.798	(0.741–0.854)	<0.001	0.99	0.754	0.748	0.502
GRACE score ≥ 89	0.781	(0.737–0.826)	<0.001	0.90	0.672	0.786	0.457
MetS	0.627	(0.569–0.685)	<0.001	1.01	0.440	0.757	0.197
MACEs	0.723	(0.664–0.783)	<0.001	0.87	0.816	0.497	0.313
Cardiac death	0.703	(0.595–0.811)	0.006	0.89	0.875	0.485	0.360
Non-fatal MI	0.676	(0.558–0.793)	0.008	1.03	0.550	0.736	0.286
TVR	0.699	(0.609–0.790)	0.001	0.82	0.958	0.369	0.328
Heart failure	0.708	(0.567–0.848)	0.019	0.96	0.727	0.630	0.357

*Abbreviations.* Cys C: cystatin C; CRP: C-reaction protein; BNP: B-type natriuretic peptide; MetS: metabolic syndrome; MACEs: major adverse cardiovascular events; MI: myocardial infarction; TVR: target vessel revascularization.

**Table 5:** Univariate and multivariate logistic regression analyses for metabolic syndrome.

	Univariate			Multivariate		
	OR	95% CI	*P* value	OR	95% CI	*P* value
Age	0.999	(0.979–1.019)	0.901			
Male	1.516	(0.965–2.381)	0.071	1.181	(0.571–2.442)	0.654
BMI	1.41	(1.291–1.541)	<0.001	1.378	(1.242–1.530)	<0.001
Smoking	1.529	(1.013–2.310)	0.043	0.932	(0.498–1.744)	0.825
Hypertension	2.317	(1.504–3.570)	<0.001	2.541	(1.438–4.489)	0.001
Diabetes	4.413	(2.834–6.872)	<0.001	3.208	(1.651–6.232)	0.001
FBG	1.278	(1.151–1.419)	<0.001	1.07	(0.921–1.243)	0.379
TG	1.648	(1.365–1.991)	<0.001	1.277	(1.030–1.584)	0.026
LDL-C	1.132	(0.879–1.457)	0.337			
HDL-C	0.048	(0.018–0.133)	<0.001	0.068	(0.018–0.260)	<0.001
High cystatin C	1.944	(1.280–2.952)	0.002	2.299	(1.251–4.225)	0.007
Uric acid	1.005	(1.003–1.007)	<0.001	1.001	(0.997–1.004)	0.659
eGFR-MDRD	0.989	(0.980–0.999)	0.035	0.993	(0.978–1.007)	0.312
CRP	1.012	(0.998–1.026)	0.096	0.996	(0.979–1.014)	0.682
D-dimer	1.005	(0.866–1.166)	0.948			
Fibrinogen	0.993	(0.971–1.016)	0.566			

High cystatin C is defined as cystatin C concentration > 0.90 mg/L.

*Abbreviations.* BMI: body mass index; FBG: fasting blood glucose; TG: triglyceride; LDL-C: low-density lipoprotein cholesterol; HDL-C: high-density lipoprotein cholesterol; eGFR-MDRD: estimated glomerular filtration rate based on MDRD equation; CRP: C-reaction protein.

**Table 6:** Association between Cystatin C and cardiovascular outcomes.

		Unadjusted			Adjusted model 1			Adjusted model 2	
Outcome variables	HR	95% CI	*P* value	HR	95% CI	*P* value	HR	95% CI	*P* value
MACEs	2.876	(1.739–4.756)	<0.001	2.677	(1.566–4.576)	<0.001	2.609	(1.295–5.257)	0.007
Cardiac death	4.780	(1.362–16.778)	0.015	4.147	(1.097–15.672)	0.036	4.400	(0.845–22.896)	0.078
Non-fatal MI	2.036	(0.812–5.106)	0.129	2.373	(0.887–6.346)	0.085	4.041	(1.023–15.964)	0.046
TVR	2.649	(1.098–6.390)	0.030	2.351	(0.912–6.062)	0.077	1.563	(0.426–5.731)	0.500
Heart failure	4.955	(1.070–22.942)	0.041	3.000	(0.589–15.293)	0.186	2.958	(0.355–24.633)	0.316
Non-fatal stroke	1.677	(0.280–10.043)	0.571	1.750	(0.281–10.894)	0.548	1.787	(0.111–28.837)	0.682

High cystatin C is defined as cystatin C concentration > 0.90 mg/L.

Univariate and multivariate Cox proportional regression analyses are applied.

Model 1 is adjusted for age, gender, BMI, smoking, hypertension, diabetes, LDL-C, and HDL-C.

Model 2 is adjusted for model 1, eGFR-MDRD, CRP, Killip class > 1, GRACE score, SYNTAX score, ACEI/ARB, beta-blocker, and PCI/CABG.

*Abbreviations.* MACEs: major adverse cardiovascular events; MI: myocardial infarction; TVR: target vessel revascularization.

## Data Availability

The data used to support the findings of this study are available from the corresponding author upon request.
